# Enhancement of *β*-Mannanase Production by *Bacillus subtilis* ATCC11774 through Optimization of Medium Composition

**DOI:** 10.3390/molecules25153516

**Published:** 2020-07-31

**Authors:** Nor Amalina Binti Mahamad Norizan, Murni Halim, Joo Shun Tan, Sahar Abbasiliasi, Miskandar Mat Sahri, Firdaus Othman, Arbakariya Bin Ariff

**Affiliations:** 1 Bioprocessing and Biomanufacturing Research Centre, Faculty of Biotechnology and Biomolecular Sciences, Universiti Putra Malaysia UPM, Serdang 43400, Selangor, Malaysia; amal88biotech@gmail.com (N.A.B.M.N.); murnihalim@upm.edu.my (M.H.); 2 Department of Bioprocess Technology, Faculty of Biotechnology and Biomolecular Sciences, Universiti Putra Malaysia UPM, Serdang 43400, Selangor, Malaysia; 3 Bioprocess Technology, School of Industrial Technology, Universiti Sains Malaysia, Gelugor 11800, Pulau Pinang, Malaysia; jooshun@usm.my; 4 Halal Products Research Institute, Universiti Putra Malaysia UPM, Serdang 43400, Selangor, Malaysia; sahar@upm.edu.my; 5 Protein and Food Technology Unit, Product Development and Advisory Services Department, Malaysia Palm Oil Board, Bangi 43600, Selangor, Malaysia; mmatsahri@gmail.com; 6 Milling and Processing Unit, Engineering and Processing Division, Malaysia Palm Oil Board, Bangi 43600, Selangor, Malaysia; fidos@mpob.gov.my

**Keywords:** *β*-mannanase, *Bacillus subtilis*, palm kernel cake, optimization, response surface methodology

## Abstract

Palm kernel cake (PKC) has been largely produced in Malaysia as one of the cheap and abundant agro-waste by-products from the palm oil industry and it contains high fiber (mannan) content. The present study aimed to produce *β*-mannanase by *Bacillus subtilis* ATCC11774 via optimization of the medium composition using palm kernel cake as substrate in semi-solid fermentation. The fermentation nutrients such as PKC, peptone, yeast extract, sodium chloride, magnesium sulphate (MgSO_2_), initial culture pH and temperature were screened using a Plackett-Burman design. The three most significant factors identified, PKC, peptone and NaCl, were further optimized using central composite design (CCD), a response surface methodology (RSM) approach, where yeast extract and MgSO_2_ were fixed as a constant factor. The maximum *β*-mannanase activity predicted by CCD under the optimum medium composition of 16.50 g/L PKC, 19.59 g/L peptone, 3.00 g/L yeast extract, 2.72 g/L NaCl and 0.2 g/L MgSO_2_ was 799 U/mL. The validated *β*-mannanase activity was 805.12 U/mL, which was close to the predicted *β*-mannanas activity. As a comparison, commercial media such as nutrient broth, M9 and Luria bertani were used for the production of *β*-mannanase with activities achieved at 204.16 ± 9.21 U/mL, 50.32 U/mL and 88.90 U/mL, respectively. The optimized PKC fermentation medium was four times higher than nutrient broth. Hence, it could be a potential fermentation substrate for the production of *β*-mannanase activity by *Bacillus subtilis* ATCC11774.

## 1. Introduction

β-mannanase (EC 3.2.1.78) is a hemicellulytic enzyme that randomly hydrolyses internal glycosidic bonds present in a backbone chain of β-_D_-1,4-mannopyranosyl linkages of mannans and heteromannans, yielding mannooligosaccharides (MOS). It has wide applications in the bio-bleaching of pulp and paper, as a hydrolytic agent in the detergent industry, in the hydrolysis of coffee extract, in the improvement of animal feed, as a fish feed additive, as a slime control agent and in the pharmaceutical industry [1].

The production of microbial β-mannanase is promising due to its low cost, high production rate, and readily controlled conditions. Although β-mannanase is from the mannolytic group and fungi like *Sclerotium rolfsii*, *Aspergillus* species and *Rhizopus* get the most attention in β-mannanase production, bacteria such as *Bacillus subtilis* TJ-102 have been reported as potential microbial mannanase producers [2]. Among different *Bacillus* species, *Bacillus subtilis* has been shown to produce highly active β-mannanase [3].

Many mannan-based carbon sources have been used to cultivate microbial mannanase. These include pure mannans like locust bean gum, guar gam and konjac powder [2,4]. However, pure mannans increase the cost of β-mannanase production. Thus, it is pertinent to explore and develop cost effective enzyme production processes using low-cost substrates like agricultural and agro-industrial residues. Several studies have been carried out on the use of agro-industrial carbon sources such as copra meal, sugar beet molasses, palm kernel cake, carob pods and coconut residue for mannanase production [5,6,7,8].

Palm kernel cake (PKC) is an agriculture by-product produced following the extraction of oil from the fruits of palm oil. Being nutritionally rich and containing large amounts of hemicellulose (57.8% of mannan, 3.7% xylan) and cellulose (11.6%) make it suitable for cultivation of microorganisms on this by-product [9]. Therefore, the objective of this study was to enhance β-mannanase production from *Bacillus subtilis* ATCC 11774 using PKC as a carbon source by optimization of the fermentation parameters using a statistical approach. The significant variables selected from the screening design were then used in the optimization of β-mannanase production using RSM. Response surface methodology (RSM) is one of the efficient statistical methods that is useful for modelling and to analyze the effect of many factors with the minimum number of experiments and can also be used to refine the optimization of the process [10].

## 2. Results

### 2.1. Selection of Basal Medium

Results from screening of the three types of basal media for bacterial growth and β-mannanase production are shown in Table 1. The highest growth of *B. subtilis* ATCC 11774 (2.31 × 10^5^ cfu/mL) and production of β-mannanase (204.16 U/mL) were obtained in nutrient broth (NB). A great reduction in the cell concentration was observed in fermentation using M9 minimal medium (M9) as substrate. The production of β-mannanase in NB was 2.3 and 4 times higher than Luria broth (LB) and M9, respectively.

### 2.2. Effect of Different Types of Carbon Source on β-Mannnanase Production

**Figure 1:**
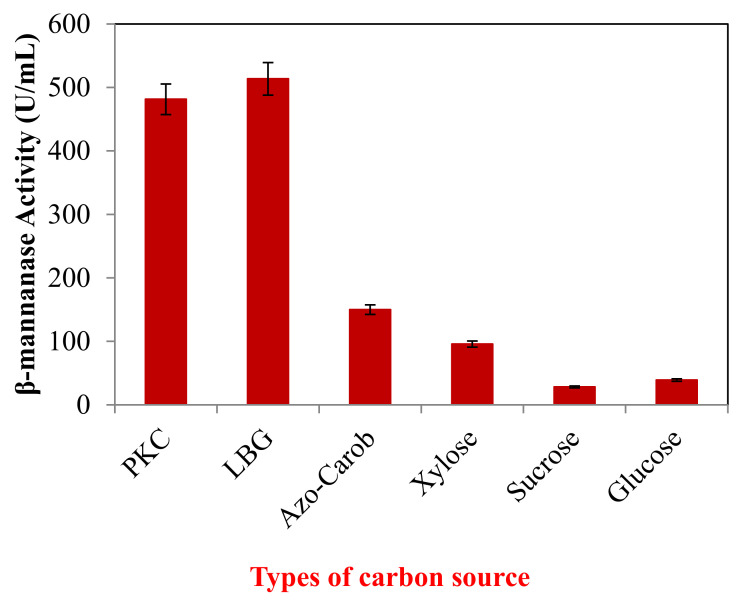
Different types of carbon sources in modified nutrient broth medium on β-mannanase production. PKC = Palm kernel cake, LBG = locust bean gum, Azo-Carob = Azo-Galactomannan from carob. The results are expressed as the means of triplicate readings with an estimated error of ± 5%.

Figure 1 shows the effect of different types of carbon sources on β-mannanase production by *B. subtilis* ATCC 11774. *B. subtilis* ATCC 11774 grew well on PKC (481.55 U/mL) and locust bean gum (LBG) (513.68 U/mL), while the production was reduced when azo-carob (150.09 U/mL), xylose (95.83 U/mL), sucrose (28.30 U/mL) and glucose (39.01 U/mL) were used as carbon source.

### 2.3. Screening of Medium Constituents Using Plackett-Burman Experimental Design

In order to optimize the medium constituents, the Plackett-Burman design was firstly used to screen the significance of individual factors. Table 2 shows the results of the experiment using the Plackett-Burman design for β-mannanase production by *B. subtilis* ATCC 11774. Results show that the growth of *B. subtilis* ATCC 11774 and production of β-mannanase were increased when a high concentration of PKC (20 g/L) was added. The highest β-mannanase production was obtained in Run#2 (658.22 U/mL) and Run#5 (649.16 U/mL), where there was a combination of PKC and peptone concentration at 20 g/L with 10 g/L of sodium chloride, at an initial pH of 6.0.

A first-order model from Equation (1) was fitted to the results obtained from the twelve experiments (Equations (1) and (2)):

Y_1_ = 593.25 + 43.30A + 16.63B − 11.13C − 22.53D − 3.83E − 11.91F − 4.53G (1)

Y_2_ = 8.98 + 0.37A + 0.16B − 0.11C − 0.26D − 0.049E − 0.12F − 0.074G (2)

where Y_1_ = β-mannanase activity (U/mL), Y_2_ = cell concentration (log [CFU/mL]), A = PKC (g/L), B = peptone (g/L). C = yeast extract (g/L), D = NaCl (g/L), E = MgSO_4_ (g/L), F = initial pH and G = temperature (°C).

Analysis of variance (ANOVA) presented the coefficient of determination (R^2^ = 0.9543) indicating that 95.43% of the variability could be explained by the model (Table 3). Factors evidencing *p* ≤ 0.05 were considered to have significant effects on the production of β-mannanase and were therefore selected for further study. ANOVA through a factorial model suggested that PKC (A), peptone (B) and sodium chloride (D) were the most significant factors greatly influencing the production of β-mannanase by *B. subtilis* ATCC 11774. Yeast extract, pH and temperature did not largely affect the production of β-mannanase in low or high concentrations of PKC. At low PKC concentration, low growth of *B. subtilis* and β-mannanase production was obtained.

### 2.4. Method of Steepest Ascent

The direction of the steepest ascent path could be determined using Equation (5). The concentrations of yeast extract (C) and magnesium sulfate (E) were fixed at 3.0 g/L and 0.2 g/L, while initial pH (F) and temperature (G) were set at pH 6 and 30 °C. The concentration of PKC (A), peptone (B) and NaCl (D) were significant factors, and the coefficients of A and B were positive, while coefficient D was negative, meaning that the increasing concentrations of A and B while decreasing the concentration of D had a positive effect on β-mannanase production. The design of the experiment of the steepest ascent and corresponding results are shown in Table 4. β-mannanase production on Step 5 was the highest (739.26 U/mL). Thus, the result of Step 5 was selected as the center point for the optimization study because these levels of concentration of PKC, peptone and NaCl were considered near the optimal region for product improvement of the fermentation process. The result from the steepest ascent also indicated that concentrations of PKC higher than 16.6 g/L would inhibit β-mannanase production.

### 2.5. Optimization of Medium Compositions Using Response Surface Methodology

Results from the full factorial composite design (CCD) for three significant factors (PKC, Peptone and NaCl concentrations) are as in Table 5. The optimal concentrations of PKC, peptone and NaCl for maximum production of β-mannanase from *B. subtilis* (790.00–802.00 U/mL) were observed at Run# 15, 16, 17, 18, 19 and 20, whereby the medium composition consisted of 16.50 g/L of PKC, 19.00 g/L of peptone and 3.00 g/L of NaCl, while the lowest production of β-mannanase was observed in Run# 1.

β-mannanase activity was described using a quadratic polynomial model. The results of the analysis of variance (ANOVA) are shown in Table 6. The values of the model were tested, and it was determined if more a complex model would have a better fit. The model has strong evidence to explain the variation in the response if the fisher-test (F-test) for the model is significant at the 5% level (*p* ≤ 0.05). The model F-value of 229.94 with *p* < 0.0001 implied that the model has been demonstrated as having a very high significance for the regression model. Hence, the lack of fit F-value of 2.82 implied that the model was not significant relative to pure error. There is a 13.97% chance that a lack of fit F-value could occur due to noise. The high F-value and non-significant lack of fit indicated that the model was a better fit.

The goodness of fit of the model was examined using the value of determination coefficient (R^2^). The value of R^2^ must be within the range of 0 ≤ R^2^ ≤ 1; a common study showed that good R^2^ should be at least 80%. The closer the R^2^ value to 1, the better the model fits to the experimental data and less is the distance between the predicted and experimental values. The value of determination coefficient (R^2^ = 0.9952) derived from β-mannanase production shows that only 0.48% of the total variation could not be explained by the model, indicating that the quadratic polynomial model was significant and sufficient to represent the actual relationship between the variables and response. Moreover, correlation between predicted R^2^ (0.9711) and adjusted R^2^ (0.9909) indicates the actual and predicted values confirmed each other and the model was reasonable and of high accuracy in predicting the values of the variables.

The model equation was found adequate to predict the interactions among the variables and the optimum concentration of the complex medium. The simplified second order regression equation for β-mannanase production as a function of concentration of PKC, peptone and NaCl was expressed as Equation (3):

Y= 797.98 + 17.96A + 12.37C − 17.51A^2^ − 39.77B² − 55.01C² − 29.06AB − 25.61AC − 10.43BC (3)

where Y is β-mannanase activity (U/mL), and A, B and C are PKC (g/L), peptone (g/L) and NaCl (g/L) concentrations, respectively.

**Figure 2:**
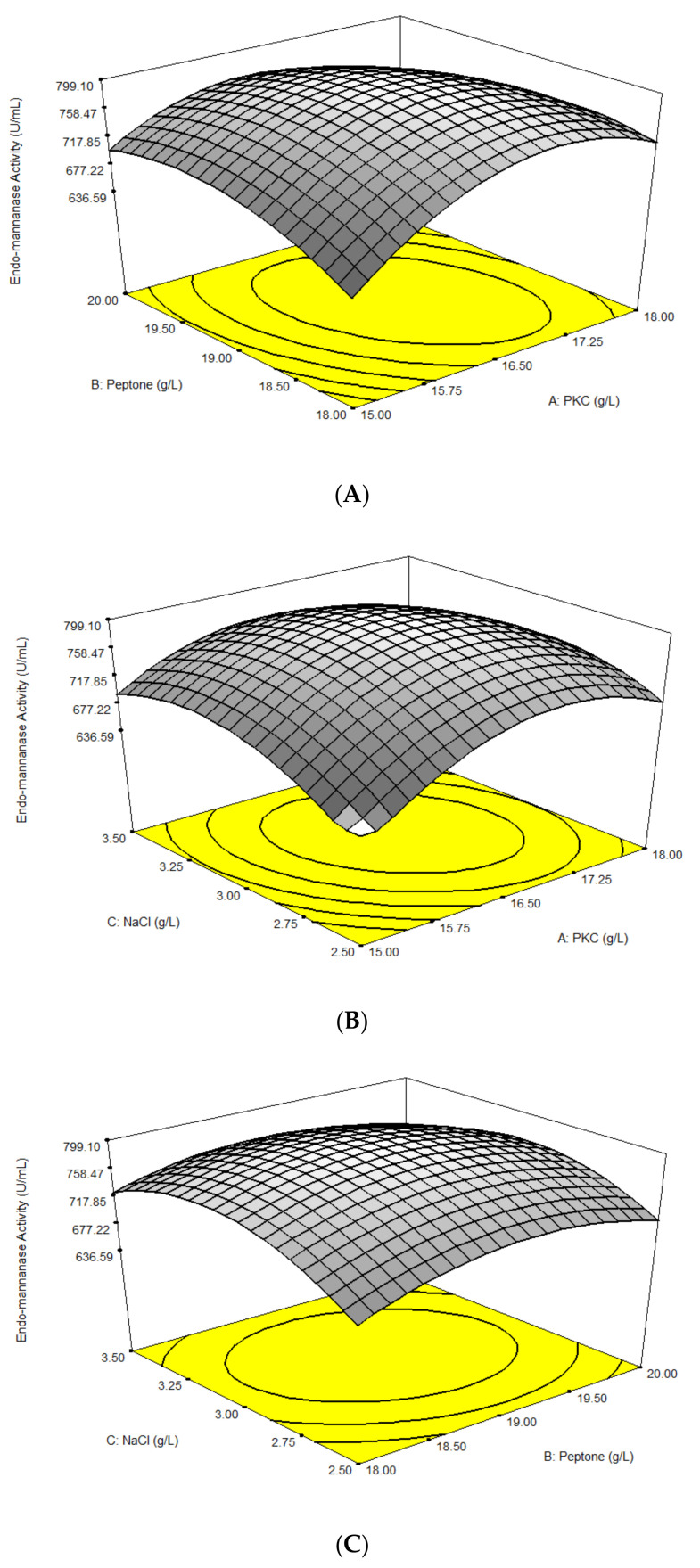
Surface plot obtained from optimization using RSM for the combination effect of (**A**) PKC concentration and peptone concentration (by keeping NaCl concentration at center point), (**B**) PKC and NaCl concentration (by keeping peptone concentration at center point) and (**C**) NaCl and peptone concentration (by keeping PKC concentration at center point) on β-mannanase activity.

Figure 2 shows the three-dimensional (3D) response surface model graphs resulted from interaction of three factors such as PKC, peptone and sodium chloride concentrations. The results illustrated that the mannanase concentration increased with an increase in intermediate levels of PKC and peptone concentrations (Figure 2A). The interaction effect between PKC and peptone concentrations was the most significant (*p* ≤ 0.0001). However, excess concentration led to slightly decreased β-mannanase production. The optimum β-mannanase production was observed with 16.5 g/L PKC and 19.0 g/L peptone. The effect of interaction between PKC and sodium chloride concentrations on the β-mannanase production is shown in Figure 2B. β-mannanase production increased with increase in PKC and NaCl concentrations. The interaction effect between them was highly significant (*p* ≤ 0.0001). The optimum β-mannanase production was obtained with 16.5 g/L PKC and 3.0 g/L NaCl concentrations. Figure 2C represents the interaction between sodium chloride and peptone concentrations when the concentration of PKC was kept constant. The presence of sodium chloride supported improvement in the β-mannanase production yield up to 3.0 g/L. However, at higher concentrations, it did not support enzyme production. The interaction effect between PKC and sodium chloride was significant (*p* ≤ 0.0050).

### 2.6. Validation of Predicted Fermentation Parameters

**Figure 3:**
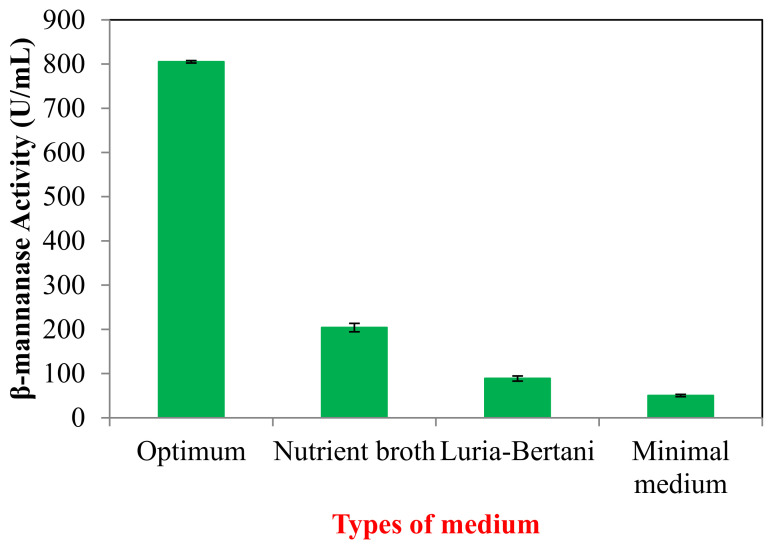
Verification experiment for β-mannanase by *B. subtilis* ATCC 11774. The results are expressed as the means of triplicate readings with an estimated error of ± 10%.

In order to validate the obtained data and to evaluate the accuracy of the applied Plackett–Burman and CCD statistical design, a verification experiment was carried out to predict the near optimum levels of independent variables. Based on the result obtain from CCD, the optimized medium composition with 16.50 g/L PKC, 19.59 g/L peptone, 3.00 g/L yeast extract, 2.72 g/L NaCl and 0.2 g/L MgSO_4_ was selected. The data were compared to minimal medium (M9), NB and LB. As observed in Figure 3, β-mannanase production was increased ~4 times higher than original NB medium when growing in optimized medium.

### 2.7. Comparison of β-Mannanase Production by Bacillus subtilis ATCC 11774 in Shake Flask and 2 L Stirred Tank Bioreactor

**Figure 4:**
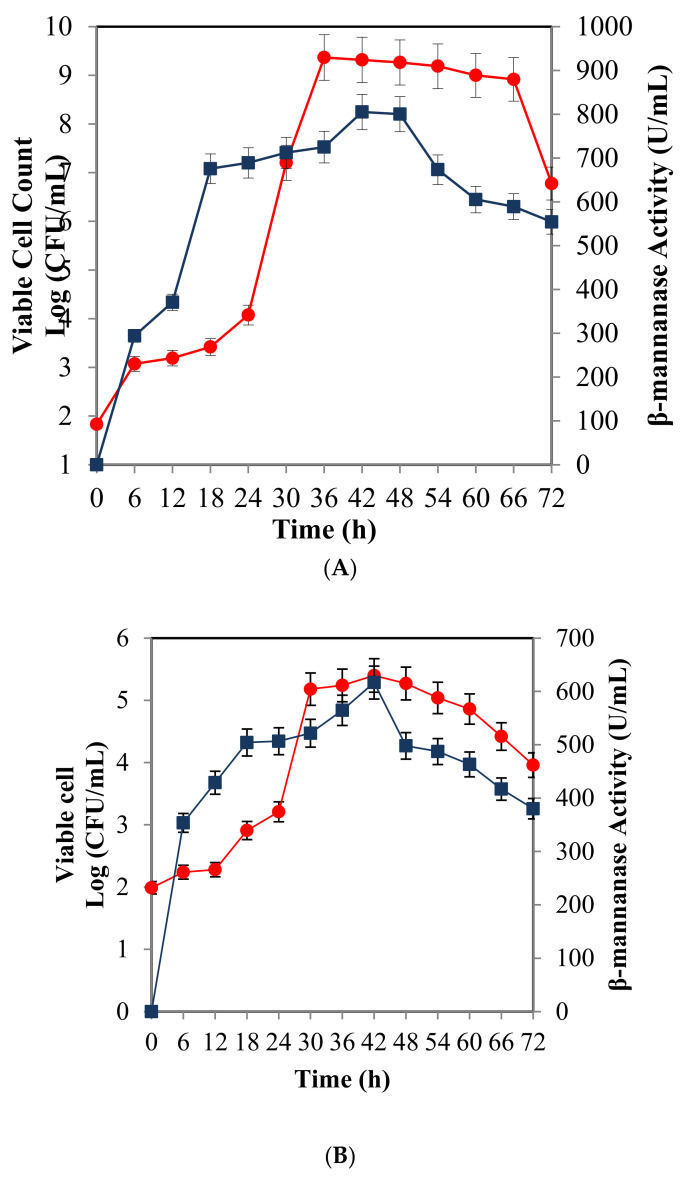
Growth of *B. subtilis* ATCC 11774 and β-mannanase production in a shake flask (**A**) and 2-L bioreactor (**B**) using optimized medium. Symbols: (●) growth of *B. subtilis* ATCC 11774; (■) β-mannanase activity. The results are expressed as the means of triplicate readings with an estimated error of ±5%.

The time course of β-mannanase production by *B. subtilis* in a shake flask is shown in Figure 4A. An exponential growth phase was observed between 12 to 36 h of fermentation. Growth reached a stationary phase after 36 h, followed by a death phase after 66 h. The β-mannanase production increased drastically between 6 to 42 h and reached the highest at 42 h. Substantial β-mannanase production occurred concomitantly with increasing bacterial growth, and β-mannanase production ended when growth reached the stationary phase. The typical time course of *β-mannanase* production by *B. subtilis* in a 2 L stirred tank bioreactor using optimized medium is presented in Figure 4B. The growth of *B. subtilis* increased rapidly between 24 to 30 h. The stationary phase was between 48–60 h and the β-mannanase activity reached maximum activity (616.73 U/mL) at 42 h. After 60 h, the growth decreased and entered the death phase.

Table 7 shows the results obtained from kinetic studies of cell growth and β-mannanase production by *B. subtilis* ATCC 11774. Based on the result, the maximum specific growth rate (µ_max_) for *B. subtilis* ATCC 11774 in a shake flask (0.523 h^−1^) was higher than the specific growth rate in a bioreactor (0.3672 h^−1^). The maximum β-mannanase activity (P_max_) for both batch fermentations was attained during the stationary phase, reaching values as high as 805.12 and 616.73 U/mL after 42 h in a shake flask and 2 L bioreactor, respectively.

## 3. Discussion

In spite of the ability to produce competitive amounts in the production of β-mannanase using the investigated method of submerged fermentation (SmF), production was affected by a variety of physicochemical factors. Liquid cultures were used in this research because it is easier to control the environmental factors required for the optimum growth of *B. subtilis* and β-mannanase production and it also allows control over the agitation speed, pH and temperature of the medium. Although solid state fermentation has more advantages for agricultural residues, submerged fermentation, however, is a preferred method for the production of extracellular enzymes.

In this study, several carbon sources consisting of LBG, azo-carob, xylose, glucose and sucrose were examined as carbon sources in comparison to that of PKC for β-mannanase production by *B. subtilis*. The complex polysaccharides, LBG and PKC, were found to induce the production of β-mannanase and were consumed by *B. subtilis* for growth and cell maintenance. This is similar to the study of Ademark et al. (1998), which reported that the addition of galactomannan (locust bean gum) to the culture media induced formation of β-mannanase [11]. This study focused on the formulation of an economical medium for β-mannanase production utilizing inexpensive substrates, and among all the tested media, PKC remained the best substrate to enhance β-mannanase production. It is desirable for the material used in the fermentation medium to be of low cost, available in large amounts and continually renewable.

Conventional methods in fermentation optimization require each factor to be treated separately, which is laborious, incomplete and time consuming [12]. RSM, being a non-conventional approach, is a collection of statistical and mathematical methods for designing experiments, developing models, evaluating the effects of factors and searching for optimum conditions and can be used to quantify the interaction between different factors. This approach provides statistically reliable results with fewer numbers of experiments and is very useful for the development, improvement and optimization of the biomanufacturing processes [13]. RSM is based on analysis of responses induced by specific factors. Using RSM in this study, we could simplify the optimization process and reduce the production costs. In addition, the most significant factors that influenced the response to the greatest extent were also determined. In this work, a high degree of similarity was observed between the predicted and experimental values, which reflected the accuracy and applicability of RSM to optimize the process for enzyme production. Similar improved production was reported in other RSM experiments, most notably in the case of β-mannanase from *Bacillus subtilis* TJ-102 and endo-mannanase production by *Bacillus* sp. CFR1601 [2,3].

An experiment using central composite design was performed to optimize the medium component concentrations. Among the three significant factors tested, palm kernel cake concentration gave the biggest effect compared with peptone and NaCl concentration. This result indicates that the palm kernel cake concentration is an efficient inducer for β-mannanase production, as it contains in its composition a high amount of soluble reducing sugar, required for the initiation of growth and replication of hemicellulose (inducer of β-mannanase), and an organic nitrogen source, necessary for protein biosynthesis. The addition of bactopeptone as an organic nitrogen source can lead to an increase in *B. subtilis* growth and β-mannanase production. Based on statistical analysis through Plackett-Burman and CCD, the concentration bactopeptone also has a significant effect on mannanase production. Instead of bactopeptone, yeast extract was also utilized as a nitrogen source in this study. A study conducted by Teodoro and Martins [14] showed yeast extract and peptone is favored for the growth and synthesis of α-amylase by *Bacillus licheniformis* SPT 27. Bactopeptone consists of a higher protein content than other organic and inorganic nitrogen sources [15]. In other findings, bactopeptone has been reported to be the best nitrogen source to enhance mannanase production by *Aspergillus niger* and *Bacillus nealsonii* PN-11 [16,17]. In the present work, an increase in β-mannanase production was comparable with those observed by Srivastava and Kapoor [3] and even higher than that of Zurmiati et al. [5].

The growth of *B. subtilis* and β-mannanase activity in a bioreactor is lower than in a shake flask. In a shake flask, the highest cell concentration (X_max_) in the experimental data was 3.02 × 10^9^ CFU/mL after 42 h with a maximum cell productivity of 8.40 × 10^10^ cells/L/h, while the cell concentration (X_max_) and cell productivity were 2.82 × 10^5^ CFU/mL and 6.70 × 10^6^ cells/L/h in bioreactor fermentation, respectively. The maximum β-mannanase activity (P_max_) for both fermentation processes was attained during the stationary phase, reaching values as high as 805.12 U/mL in a shake flask and 616.73 U/mL in a 2 L bioreactor, respectively. A previous study using *B.* *nealsonii* PN-11 on minimal media showed that the highest β-mannanase concentration was obtained in the stationary phase [17]. A similar finding has been reported by Mubarak et al. [18] in a study of the relationship between cell growth and surfactin production; the highest surfactin production by *B. subtilis* ATCC 21,332 and *B. subtilis* MSH1 was reached at the stationary phase. Further fermentation process demonstrated a decline in cell mass and enzyme production due to random lethal events, which caused cellular fragmentation in the death phase and released intracellular toxic materials into the fermentation broth [19]. Based on the findings of this study, bacterial growth in the shake flask declined drastically from 66 h, while in the bioreactor, bacterial growth started to decrease after 48 h.

The culture conditions in the shake flask and bioreactor are quite different because the agitation speeds, aeration rates and incubation temperatures also influence the performance of growth and enzyme production. Substantial decrease in β-mannanase activity with prolonged fermentation might be due to the degradation of the protein or leakage of the protein into the medium. However, production of β-mannanase in a 2 L bioreactor using optimized culture medium could be improved, which is interesting for industrial applications.

## 4. Conclusions

Results from this study have demonstrated the importance of submerged fermentation for the production of enzyme using palm kernel cake as a substrate, which offers significant benefit due to its abundant availability and low cost. Statistical optimization of the fermentation process using RSM was successfully used to enhance β-mannnase production by *B. subtilis*. The Plackett-Burman design pre-selected the significant factors such as PKC, peptone and NaCl. The medium formulation was further optimized using CCD where the most significant factors for enhancing β-mannanase by *B. subtilis* were identified. The model equation was found adequate to predict the interactions among the variables and optimum concentration of the complex medium. The highest β-mannanase activity (805.12 U/mL) was obtained in optimized medium, which consisted of 16.50 g/L PKC, 19.59 g/L peptone, 3.00 g/L yeast extract, 2.72 g/L NaCl and 0.2 g/L MgSO4. Results from this study provided useful information for the optimization of medium composition for the other enzyme production through fermentation processes.

## 5. Materials and Methods

### 5.1. Bacterial Strain, Medium and Inoculum Preparation

*Bacillus subtilis* ATCC 11774 was used in this study. PKC was obtained from FELDA Kernel Product Sdn. Bhd., Negeri Sembilan, Malaysia. PKC was ground using a blender and sieved using mesh to a particle size of 2 mm and dried in an oven at 60 °C for 48 h. Primary culture was prepared by taking a single bacterial colony from a nutrient agar (NA) plate and grown in 10 mL nutrient broth (NB) at 30 °C for 12 h. This initial culture was inoculated into 500 mL baffled shake flasks containing 100 mL NB and incubated at 30 °C in an incubator shaker (INFORS Bottmingen, Switzerland) with a constant shaking of 200 rpm for 12 h and used as a standard inoculum for all fermentations

### 5.2. Fermentation

Fermentations were carried out in 250 mL baffled flasks containing 50 mL of the commercial medium, namely minimal medium (M9; Merck, Darmstadt, Germany), Luria bertani (LB; Merck, Darmstadt, Germany) and nutrient broth (NB; Merck, Darmstadt, Germany), which was inoculated with 8% (*v/v*) inoculum, and incubated in an incubator shaker at 30 °C and agitated at 200 rpm for 72 h. Mean composition (g/L) of commercial culture media was as detailed in Table 1.

### 5.3. Influence of Carbon Sources on β-Mannanase Production by B. subtilis ATCC 11,774

In a preliminary experiment, six different types of carbon source including PKC, locust bean gum (LBG), azo-carob, xylose, sucrose and glucose were investigated using a multi-factor experimental design (change-one-factor-at-a-time) approach. Culture medium was prepared by mixing 1.0 g/L of each carbon source with peptone (15.0 g/L), yeast extract (3.0 g/L), NaCl (3.0 g/L) and MgSO_2_ (0.02 g/L). Medium was autoclaved at 121 °C for 15 min before fermentation.

### 5.4. Optimisation of β-Mannanase Production by B. subtilis ATCC 11,774 Using Response Surface Methodology

The statistical design and analysis were performed using Design Expert software version 6.0.67 (Stat-Ease, Inc., Minneapolis, MN, USA). Plackett–Burman experimental design was used for initial screening of the important medium components with respect to β-mannanase production. After the screening process, a method of steepest ascent was applied to move the selected medium components rapidly to the general vicinity of the optimum via experimentation. A full factorial CCD was applied to optimize the significant variables with a new operating region.

### 5.5. Initial Screening of the Important Medium Component Using Plackett-Burman

Plackett-Burman design was used to screen important factors that affect β-mannanase production by *B. subtilis* ATCC 11774. Seven numerical variables, which were PKC (10 to 20 g/L), peptone (10 to 20 g/L), yeast extract (1 to 10 g/L), NaCl (1 to 10 g/L), MgSO_2_ (0.1 to 0.5 g/L), initial pH (6 to 8) and temperature (25 to 35 °C), were selected for the screening purpose. Twelve experiments were run in triplicates with two levels of each factor for the screening in one block. A fitted first-order model is expressed as Equation (4):

(4) $$Y=\beta_{0}+\overset{k}{\underset{i=1}{\sum }}\beta_{i}x_{i}$$

where *Y* is the response (β-mannanase activity, U/mL), *x_i_* is the coded independent variable. β_0_ and β*_i_* are constant coefficients and linear coefficients of each factor, respectively. The factors whose confidence level was higher than 99% (*p* ≤ 0.05) were considered as the important medium components that have significant effect on the production of β-mannanase.

### 5.6. Method of Steepest Ascent

To move rapidly towards the neighborhood of the optimum response, the method of steepest ascent was used. New units were determined according to the estimated coefficient ratio from the first-order model in Equation (1). To move away from the first design center along the path of steepest ascent, three significance factors from the Plackett-Burman analysis (PKC, peptone and NaCl concentration) were selected. Other components were at the constant level. Increment was direct ration to regression coefficients β*_i_*. Experiments were performed along the steepest ascent path until the response did not increase anymore; this point would be near the optimal point and could be used as the center point for further optimization.

### 5.7. Optimization by Response Surface Methodology

A full factorial CCD was applied to optimize the significant variables. In the CCD, three independent factors (PKC, peptone and NaCl concentrations) were investigated at five different levels (−α, −1, 0, +1, and +α) and twenty experiments were designed, as shown in Table 2.

A second-order polynomial model (Equation (5)) was then used to describe the effects of the significant factors on the activity of β-mannanase:

(5) $$Y=\beta_{0}+\sum \beta_{i}x_{i}+\sum \beta_{ii}x_{i}+\sum \beta_{ij}x_{i}x_{j}\;\left(i,j=1,2,3\;\mathrm{and}\;i\neq j\right)$$

where Y was the estimated response (enzyme activity, U.mL^−1^); X_1_, X_2_ and X_3_ were the concentration (g/L) of PKC, peptone and NaCl, respectively. *β*_*0*_ and *β*_*i*_ were linear coefficients of each factor, and *β*_*i*_ and *β*_*ii*_ were the quadratic coefficients. Statistical parameters were estimated using analysis of variance (ANOVA). Validation of the predicted optimum parameters was carried out with a single observation method.

### 5.8. Production of β-Mannanase in 2-L Bioreactor

A 2 L bioreactor (BDCU, B. Braun, Germany) containing 1 L of optimized medium was used for the production of β-mannanase. The pH system consisted of a central module and sterilizable pH probe (Ingold). pH was allowed to fluctuate freely and monitored continuously. The dissolved tension (DOT) level was measured with a sterilizable DO electrode. The bioreactor was sterilized at 121 °C for 30 min. The fermentation medium was inoculated with 8% (*v/v*) inoculum and carried out at pH 7.0 and 30 °C with an agitation speed of 200 rpm and aeration rate of 0.1 vvm. During the fermentation, samples were taken at time intervals for the analysis of β-mannnanase activity and the number of viable cells.

### 5.9. Analytical Procedures

The mannanase assay was carried out according to Aziz et al. [1] with some modification. β-mannanase activity was assayed using 0.5% (*w/v*) locust bean gum (LBG) from Ceratonia siliqua seeds (Sigma-Aldrich, St. Louis, MO, USA)) as a substrate. The assay mixture contained 1.8 mL 0.5% (*w/v*) of LBG in 0.05 M citrate-phosphate buffer at pH 7 and 0.2 mL of suitably diluted supernatant of crude enzyme. The reaction mixture was incubated at 50 °C for 10 min in a water bath with moderate agitation. Reducing sugars produced due to enzyme activity were determined as mannose reducing equivalents using the modified dinitrosalicylic acid (DNS) method [20]. One unit of β-mannanase activity was defined as the amount that the enzyme releases: 1 µmole of mannose released per minute of reaction time under the assay condition used. A standard curve was plotted by using mannose as the standard solution. The β-mannanase activity was calculated using Equation (6):

(6) $$\mathrm{Mannanase}\;\left(\frac{U}{\mathrm{mL}}\right)=\frac{\left(\mathrm{final}\;\mathrm{absorbance}\right)-c}{m}\times \frac{\mathrm{dilution}\;\mathrm{factor}}{\mathrm{sample}\;\mathrm{volume}}\times \frac{1}{\mathrm{reaction}\;\mathrm{time}}\times \frac{1000\mu g}{1\mathrm{mg}}\times \frac{1\;\mu \mathrm{mole}}{180.2\;\mu g}$$

where m is the slope and c is the intercept.

The viable cell determination was carried out using colonies forming units (CFU) counting on plates as the standard method. In brief, 1 mL of diluted mixture with dilutions from 10^−4^ to 10^−10^ was transferred to nutrient agar plates to perform a plate count of bacteria using the drop plate method. The culture plates were incubated at 30 °C for 24 h. After 24 h, the colonies on each plate were counted. The colony forming unit that represents the number of viable bacterial cells per milliliter of sample was calculated using Equation (7):

(7) $$\mathrm{Number}\;\mathrm{of}\;\mathrm{bacteria}\;\left(\mathrm{mL}\right)=\frac{\mathrm{number}\;\mathrm{of}\;\mathrm{colonies}\;\left(\mathrm{CFUs}\right)}{\mathrm{dilution}\;x\;\mathrm{amount}\;\mathrm{plated}\;\left(\mathrm{mL}\right)}$$

## Figures and Tables

**Table 1:** Mean composition (g/L) and production of β-mannanase by *B. subtilis* ATCC 11,774 in commercial culture media.

	M9	NB	LB
	(g/L)	(g/L)	(g/L)
**Total sugar (TS)**			
Glucose	20	1	4
**Total nitrogen (TN)**			
Peptone (10–12% of TN)	0	15	0
Yeast extract (10.6% of TN)	0	3	5
Meat extract (12% of TN)	0	0	0
Tryptone	0	0	10
Ammonium sulphate	1	0	0
NaCl	0.5	3	10
Na_2_HPO4	12.5	0	0
KH_2_PO4	3	0	0
MgSO_2_	2	0	0
CaCl_2_	1	0	0
Final cell concentration	1.12 × 10^2^	2.31 × 10^5^	1.04 × 10^4^
(CFU/mL)			
β-mannanase activity	50.32 ± 3.45	204.16 ± 9.21	88.90 ± 7.08
(U/mL)			

**Table 2:** Screening of influencing factor on β-mannanase production using Plackett-Burman.

Std	Experimental Variables							Dummy Variables					
	X_1_	X_2_	X_3_	X_4_	X_5_	X_6_	X_7_	X_8_	X_9_	X_10_	X_11_	Enzyme	Cell Concentration
	PKC (g/L)	peptone (g/L)	yeast extract (g/L)	NaCl (g/L)	MgSO_4_ (g/L)	Initial pH *	Temperature (° C)					Activity (U/mL)	Log (CFU/mL)
1	20.0 (+1)	10.0 (−1)	10.0 (+1)	1.0 (−1)	0.1 (−1)	6.0 (−1)	35.0 (+1)	(+1)	(+1)	(−1)	(+1)	639.91	9.38
2	20.0 (+1)	20.0 (+1)	1.0 (−1)	10.0 (+1)	0.1 (−1)	6.0 (−1)	25.0 (−1)	(+1)	(+1)	(+1)	(−1)	658.22	9.47
3	10.0 (−1)	20.0 (+1)	10.0 (+1)	1.0 (−1)	0.5 (+1)	6.0 (−1)	25.0 (−1)	(−1)	(+1)	(+1)	(+1)	606.93	9.27
4	20.0 (+1)	10.0 (−1)	10.0 (+1)	10.0 (+1)	0.1 (−1)	8.0 (+1)	25.0 (−1)	(−1)	(−1)	(+1)	(+1)	602.46	9.10
5	20.0 (+1)	20.0 (+1)	1.0 (−1)	10.0 (+1)	0.5 (+1)	6.0 (−1)	35.0 (+1)	(−1)	(−1)	(−1)	(+1)	649.16	9.46
6	20.0 (+1)	20.0 (+1)	10.0 (−1)	1.0 (−1)	0.5 (+1)	8.0 (+1)	25.0 (−1)	(+1)	(−1)	(−1)	(−1)	637.01	9.35
7	10.0 (−1)	20.0 (+1)	10.0 (+1)	10.0 (+1)	0.1 (−1)	8.0 (+1)	35.0 (+1)	(−1)	(+1)	(−1)	(−1)	503.62	8.08
8	10.0 (−1)	10.0 (−1)	10.0 (+1)	10.0 (+1)	0.5 (+1)	6.0 (−1)	35.0 (+1)	(+1)	(−1)	(+1)	(−1)	502.78	8.02
9	10.0 (−1)	10.0 (−1)	1.0 (−1)	10.0 (+1)	0.5 (+1)	8.0 (+1)	25.0 (−1)	(+1)	(+1)	(−1)	(+1)	508.10	8.15
10	20.0 (+1)	10.0 (−1)	1.0 (−1)	1.0 (−1)	0.5 (+1)	8.0 (+1)	35.0 (+1)	(−1)	(+1)	(+1)	(−1)	632.54	9.30
11	10.0 (−1)	20.0 (+1)	1.0 (−1)	1.0 (−1)	0.1 (−1)	8.0 (+1)	35.0 (+1)	(+1)	(−1)	(+1)	(+1)	604.31	9.16
12	10.0 (−1)	10.0 (−1)	1.0 (−1)	1.0 (−1)	0.1 (−1)	6.0 (−1)	25.0 (−1)	(−1)	(−1)	(−1)	(−1)	573.94	8.96

*Initial pH refers to the pH of citrate-phosphate buffer added to the sterile medium.

**Table 3:** ANOVA of Plackett-Burman screening design experiment for the production of β-mannanase.

Source	Sum of Squares	*df*	Mean Square	F Value	*p*-Value
**Y_1_: Enzyme Activity**					
Model	35,516.44	7	5073.78	11.92	0.0153 ^a^
PKC, (A)	22,500.41	1	225,000.41	52.87	0.0019 ^a^
Peptone, (B)	3317.35	1	3317.35	7.79	0.0492 ^a^
Yeast extract, (C)	1486.52	1	1486.52	3.49	0.1350 ^b^
NaCl, (D)	6088.51	1	6088.51	14.31	0.0194 ^a^
MgSO_4_, (E)	175.87	1	175.87	0.41	0.5553 ^b^
Initial pH, (F)	1701.70	1	1701.70	4.00	0.1162 ^b^
Temperature, (G)	246.07	1	246.07	0.58	0.4894 ^b^
Residual	311.48	4	425.61		
Cor Total	37,218.87	11			
**Y_2_: Cell Concentration**					
Model	3.16	7	0.45	6.61	0.0436 ^a^
PKC, (A)	1.64	1	1.64	23.95	0.0081 ^a^
Peptone, (B)	0.30	1	0.30	4.36	0.1051 ^b^
Yeast extract, (C)	0.14	1	0.14	2.09	0.2214 ^b^
NaCl, (D)	0.82	1	0.82	11.95	0.0259 ^a^
MgSO_4_, (E)	0.029	1	10.029	0.42	0.5501 ^b^
Initial pH, (F)	0.17	1	0.17	2.50	0.1893 ^b^
Temperature, (G)	0.066	1	0.066	0.97	0.3812 ^b^
Residual	0.27	4	0.068		
Cor Total	3.43	11			

^a^ Significant at *p* ≤ 0.050. ^b^ Insignificant at *p* ≥ 0.050.

**Table 4:** Path of steepest ascent for three variables for improvement of β-mannanase production by *B. subtilis* ATCC 11,774.

Step no.	PKC	Peptone	NaCl	β-mannanase Activity (U/mL)
	(g/L)	(g/L)	(g/L)	
1	15.5	16.30	4.64	518.08
2	15.75	16.95	4.20	581.97
3	16.00	17.60	3.77	628.48
4	16.25	18.35	3.34	658.20
5	16.50	19.00	3.00	799.26
6	16.75	19.55	2.48	676.95

**Table 5:** Full factorial CCD matrix for the three significant variables and experimental and predicted β-mannanase production by *B. subtilis* ATCC 11774.

Run	A	B	C	β-mannanase Activity (U/mL)	
	PKC (g/L)	Peptone (g/L)	NaCl (g/L)	Experimental	Predicted
1	15.00 (−1)	18.00 (−1)	2.50 (−1)	535.06	533.17
2	18.00 (+1)	18.00 (−1)	2.50 (−1)	680.90	678.43
3	15.00 (−1)	20.00 (+1)	2.50 (−1)	608.64	616.35
4	18.00 (+1)	20.00 (+1)	2.50 (−1)	641.98	645.35
5	15.00 (−1)	18.00 (−1)	3.50 (+1)	635.58	629.98
6	18.00 (+1)	18.00 (−1)	3.50 (+1)	682.75	672.81
7	15.00 (−1)	20.00 (+1)	3.50 (+1)	671.21	671.46
8	18.00 (+1)	20.00 (+1)	3.50 (+1)	598.37	598.03
9	13.98 (−2)	19.00 (0)	3.00 (0)	564.06	562.71
10	19.02 (+2)	19.00 (0)	3.00 (0)	618.60	623.11
11	16.50 (0)	17.32 (−2)	3.00 (0)	671.21	681.97
12	16.50 (0)	20.68 (+2)	3.00 (0)	696.65	689.04
13	16.50 (0)	19.00 (0)	2.16 (−2)	626.64	621.57
14	16.50 (0)	19.00 (0)	3.84 (+2)	654.98	663.19
15	16.50 (0)	19.00 (0)	3.00 (0)	797.97	797.98
16	16.50 (0)	19.00 (0)	3.00 (0)	801.44	797.98
17	16.50 (0)	19.00 (0)	3.00 (0)	787.56	797.98
18	16.50 (0)	19.00 (0)	3.00 (0)	796.23	797.98
19	16.50 (0)	19.00 (0)	3.00 (0)	800.32	797.98
20	16.50 (0)	19.00 (0)	3.00 (0)	804.92	797.98

**Table 6:** Analysis of variance (ANOVA) and coefficient estimate (CE) using a regression model for the optimization of β-mannanase production by *B. subtilis* ATCC 11774.

Source	Coefficient Estimate	Sum of Squares	*df*	Mean Square	F-value	*p*-Value
Model	797.98	140,200	9	15,574.94	229.94	<0.0001
PKC, (A)	17.96	4403.56	1	4403.56	65.01	<0.0001
Peptone, (B)	2.10	60.28	1	60.28	0.89	0.3677
NaCl, (C)	12.37	2090.09	1	2090.09	30.86	0.0002
A²	−72.51	75,761.64	1	75,761.64	1118.52	<0.0001
B²	39.77	22,789.55	1	22,789.55	336.46	<0.0001
C²	−55.01	43,616.64	1	43,616.64	643.94	<0.0001
AB	−29.06	6758.02	1	6758.02	99.77	<0.0001
AC	−25.61	5245.70	1	5245.70	77.45	<0.0001
BC	−10.43	869.67	1	869.67	12.84	0.0050
Residual		677.34	10	67.73		
Lack of Fit		500.14	5	100.03	2.82	0.1397
Pure Error		177.20	5	35.44		
Cor Total		140,900	19			

**Table 7:** The performance of cell growth and β-mannanase production by *B. subtilis* fermentation in shake flask and 2 L stirred tank bioreactor.

Kinetic Parameter Value	Shake Flask	2 L Stirred Tank Bioreactor
Maximum cell concentration, X_max_ (CFU/mL)	3.02 × 10^9^	2.82 × 10^5^
Maximum specific growth rate, µ_max_ (h**^−^**^1^)	0.523	0.3672
Generation time, Td (min)	79.52	113.27
Cell productivity, X_max_ (cells/L/h)	8.40 × 10^10^	6.70 × 10^6^
Maximum enzyme production, P_max_ (U/mL)	805.12	616.73
Enzyme productivity (U/mL/h)	16.77	14.68
Yield (U/g PKC)	50320	38,545
